# Out-of-hospital births and neonatal outcomes: a retrospective cohort study from a level IV NICU and review of regulatory frameworks

**DOI:** 10.3389/fped.2026.1843232

**Published:** 2026-08-28

**Authors:** Lucy Eletel, Samantha Corkum, Jessica Hazard, David Riley

**Affiliations:** 1 Pediatrics, Anne Burnett School of Medicine at Texas Christian University, Fort Worth, TX, United States; 2 Cook Children’s Medical Center, Fort Worth, TX, United States; 3 Pediatrix Medical Group of Texas — Fort Worth Neonatology, Fort Worth, TX, United States

**Keywords:** birth center, home birth, midwifery regulation, neonatal intensive care, neonatal morbidity, neonatal mortality, neonatal outcomes, out-of-hospital birth

## Abstract

Out-of-hospital births have increased in the United States, yet their neonatal safety remains debated, particularly in states with lower midwifery integration. This study aimed to describe neonatal outcomes following out-of-hospital births, compare home vs. birth center deliveries, and evaluate birth center features associated with neonatal transfers. We conducted a retrospective cohort study at a Texas Level IV NICU which included 226 neonates admitted post-out-of-hospital births (2020–2024). Analysis included electronic record derived outcomes and resource utilization, supplemented by surveys of six regional birth centers on staffing, equipment, protocols, and transfers. Birth centers had more admissions (48.7% vs. 43.8% home births), but home-born infants showed worse outcomes: median NICU LOS (11.5 vs. 5 days), prolonged antibiotics (median 2 vs. 1d), Mortality (6% vs. 1.8%). Surveys revealed substantial variability: all centers screened for GBS, but only half mandated prophylaxis; 50% had IVs; there was no access to defibrillators. Hospital distance averaged 3.15 miles (travel time ∼12 min), yet transfer delays were common due to late recognition of complications. Neonates from out-of-hospital births exhibited elevated morbidity, mortality, and healthcare utilization. Standardized safety protocols, enhanced regulatory oversight, and improved emergency preparedness are needed, especially in states like Texas with lower midwifery integration.

## Introduction

In high-income countries, planned out-of-hospital births have risen steadily due to a preference for physiologic labor and concerns over excessive medical intervention (1–3). In the United States, the proportion of such births increased from 0.87% in 2004 to 1.3% in 2017, with the most rapid growth in rural counties (2, 20, 23). Leading organizations such as the American College of Obstetricians and Gynecologists (ACOG) and the American Academy of Pediatrics (AAP) recommend hospital settings for birth, especially when complications arise (1).

The safety of out-of-hospital birth is debated. Multiple U.S. studies have reported increased neonatal risks, including seizures, hypoxic-ischemic encephalopathy (HIE), sepsis, and death, even after risk adjustment (4–6, 29, 30). Additional analyses have documented elevated rates of neurologic injury, delayed newborn screening, prolonged respiratory support, and higher healthcare utilization following transfer from out-of-hospital settings (7–11). These findings have been corroborated in prospective cohorts and recent state-level analyses (7, 11, 12, 34).

Nationally, regulation of freestanding birth centers varies considerably. While most states require licensure, others rely on voluntary accreditation or lack formal oversight (13, 14). Significant interstate variation exists in provider licensure, facility standards, insurance coverage, and integration with hospital systems (15, 16, 25–28).

Texas maintains regulations broadly comparable to other large states, permitting certified nurse-midwives (CNMs), licensed midwives (LMs/CPMs), and physicians to attend low-risk out-of-hospital births while prohibiting high-risk deliveries outside the hospital (6, 21, 22). However, notable differences exist in midwife licensure pathways, facility oversight, and transfer protocols compared with states such as California and New York, which impose stricter certification and integration requirements (2, 6, 17). These regulatory and practice differences create a distinct context for evaluating neonatal outcomes after out-of-hospital birth in Texas.

The objective of this study was to evaluate neonatal outcomes among infants transferred to a Level IV NICU following planned out-of-hospital birth (home or freestanding birth center) and to examine the regulatory frameworks governing such births in Texas. Unlike prior investigations that relied primarily on large anonymized public health datasets or birth certificate registries, this study utilizes granular, patient-level clinical data from a high-acuity Level IV NICU registry, combined with a focused review of Texas regulatory requirements. This approach allows for detailed characterization of transfer indications, clinical course, and outcomes in a contemporary cohort (2020–2024) while directly contextualizing findings within the state's specific regulatory environment.

## Materials and methods

This retrospective cohort study was conducted at Cook Children's Medical Center (CCMC), a 106-bed Level IV NICU in Fort Worth, Texas. Using IRB-approved electronic medical record (EMR) queries, we identified 226 neonates who were born in out-of-hospital settings and subsequently admitted to the NICU between January 1, 2020, and December 31, 2024.

Primary outcomes included NICU length of stay (LOS) and all-cause in-hospital mortality. These outcomes were selected because they are objective, clinically meaningful indicators of neonatal risk that have been consistently associated with planned out-of-hospital birth in prior literature and are directly relevant to evaluating the safety of out-of-hospital delivery systems. Secondary measures include indicators of severe neonatal morbidity: culture-confirmed sepsis, hypoxic ischemic encephalopathy (HIE), grade ≥ III intraventricular hemorrhage, or mechanical ventilation lasting more than 48 h. These outcomes were chosen to capture severe, clinically significant morbidity events that are relatively rare but carry high risk of long-term neurodevelopmental impairment We also collected data on healthcare resource utilization, including total antibiotic days and central line days. Descriptive statistics were used to summarize demographic and clinical variables.

**Figure 1:**
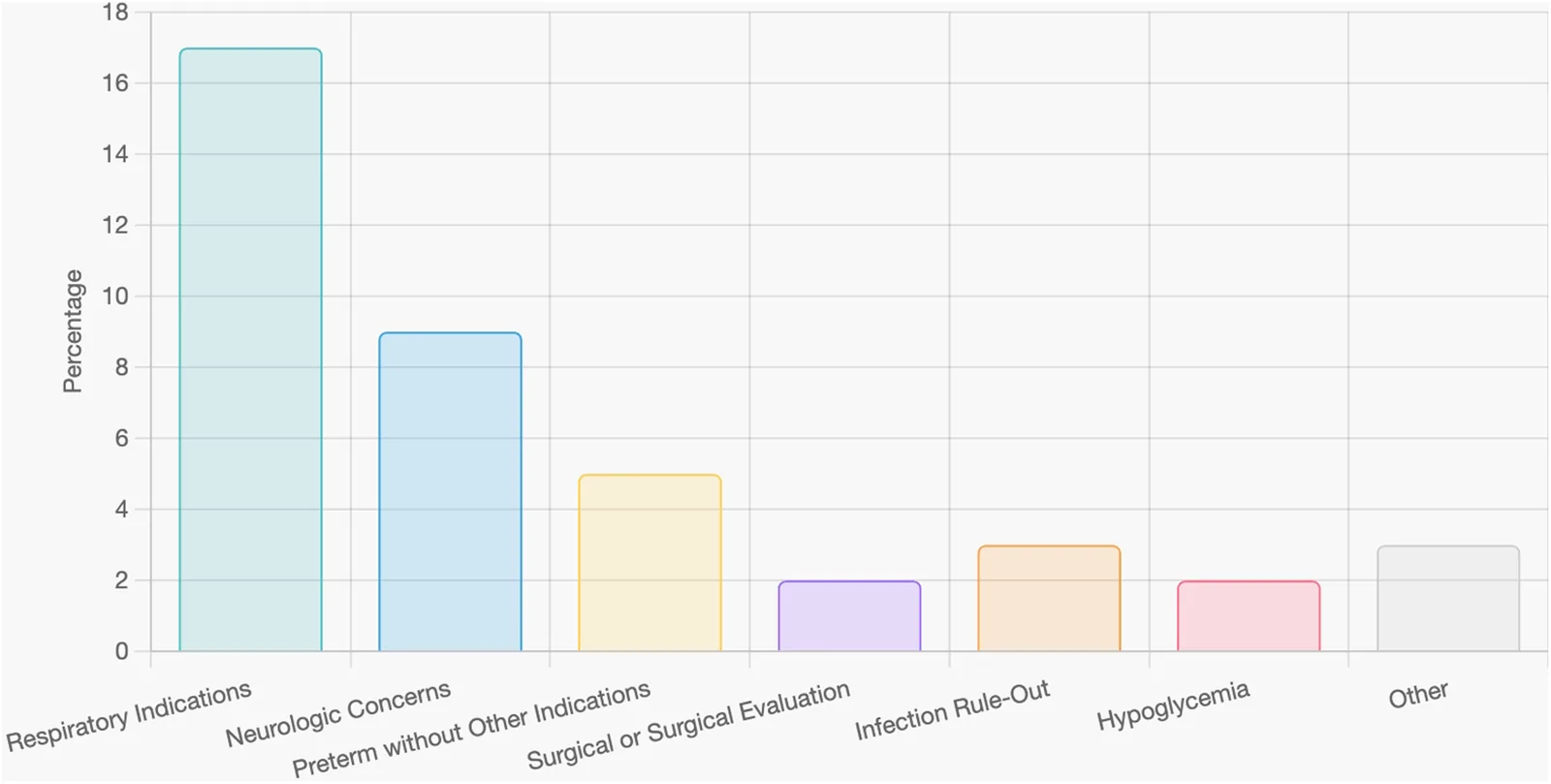
Percentage of resuscitation by referral category distribution of referral categories among neonates who required resuscitation. Respiratory indications accounted for the largest proportion of resuscitations, followed by neurologic concerns. Smaller proportions were referred for preterm birth without other indications, surgical or surgical evaluation needs, infection rule-out, hypoglycemia, and other less frequent categories.

Referral indications for NICU transfer and resuscitation were categorized into seven groups based on the primary reason documented in the transfer record and admission note: (1) Respiratory Indications—need for positive-pressure ventilation, supplemental oxygen, or respiratory support immediately after birth or during transport; (2) Neurologic Concerns—seizures, hypotonia, encephalopathy, apnea, or other acute neurologic signs prompting urgent evaluation; (3) Preterm without Other Indications—birth at <37 weeks gestation in the absence of additional complications noted at the time of transfer; (4) Surgical or Surgical Evaluation—suspected congenital anomaly, abdominal distension, or other condition requiring surgical consultation; (5) Infection Rule-Out—maternal risk factors (e.g., prolonged rupture of membranes, maternal fever, or group B Streptococcus colonization) leading to sepsis evaluation; (6) Hypoglycemia—persistent blood glucose <40 mg/dL requiring intravenous dextrose beyond standard initial management; and (7) Other—any indication not meeting the above criteria. These categories were used to classify the primary reason for resuscitation and transfer as shown in Figure 1.

To supplement clinical data and assess the local care environment, we developed and distributed structured surveys to all 13 known freestanding birthing centers operating in Tarrant County during the study period (24). These surveys aimed to assess each center's annual birth volume, staffing ratios, proximity to the nearest hospital, available resuscitation equipment, eligibility and exclusion policies, provider training, intrapartum monitoring tools, and protocols for emergency transfer. Surveys were distributed electronically and followed by reminder emails and phone calls to maximize participation. The survey instrument was designed to assess birthing center compliance with recommendations from the American College of Obstetricians and Gynecologists (ACOG) (1) and the American Academy of Pediatrics (AAP) (18). The full survey questionnaire is provided as Supplementary Material.

Among neonates born at home, we attempted to classify births as planned or unplanned based on documentation in the transfer records and maternal history; however, this information was not consistently available. Gestational age at birth was categorized according to ACOG definitions as early term (37 0/7–38 6/7 weeks), full term (39 0/7–40 6/7 weeks), late term (41 0/7–41 6/7 weeks), and post-term (≥42 0/7 weeks). Differences in outcomes according to these standard obstetric categories were examined when sample size permitted.

This study was approved by the Institutional Review Board at Cook Children's Medical Center, and a waiver of informed consent was granted due to the retrospective nature of the data collection.

## Results

**Figure 2:**
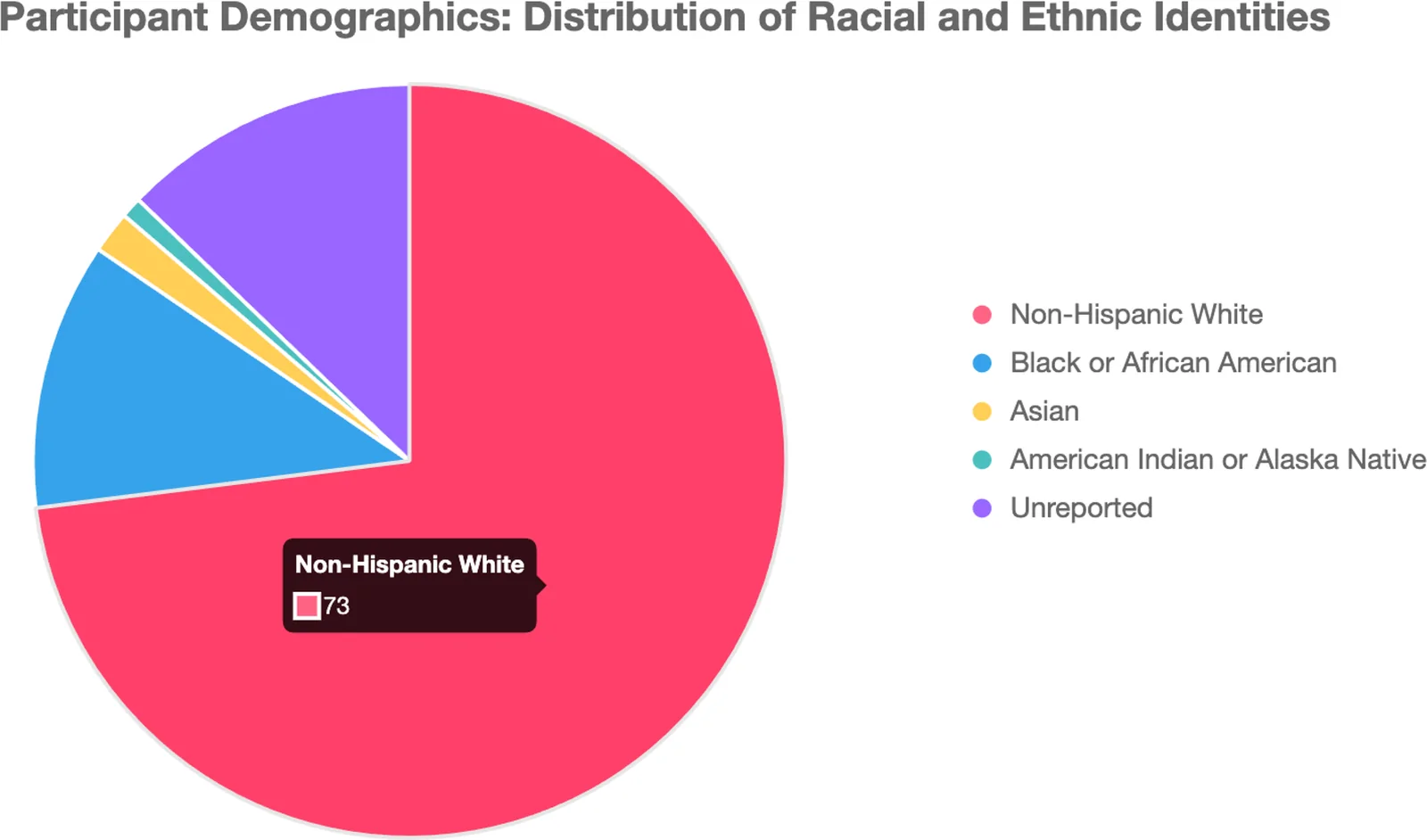
Participant demographics. Distribution of racial and ethnic identities among the study population, with the majority identifying as White, followed by smaller proportions identifying as Black or African American, Asian, American Indian or Alaska Native, or unknown/not reported.

From 2020 to 2024, a total of 5,570 infants were admitted to the NICU. Of these, 226 (4.1%) were transferred following out-of-hospital delivery. Admit sources included birth centers (48.7%, *n* = 110), home births (43.8%, *n* = 99), and emergency deliveries en route (7.5%, *n* = 17). The majority of mothers in the cohort were non-Hispanic White (73.0%), followed by Black or African American (11.5%), Asian (1.8%), and American Indian or Alaska Native (0.9%), with 12.8 percent unreported Figure 2.

The average maternal age was 29.7 years, and most births occurred at term, with 8.3% preterm. Home-born infants required greater NICU resources than those from birth centers. Among infants with available length of stay data, the median NICU length of stay was 5 (IQR 3-8) days for birth center transfers and 11.5 days (IQR 5-31.5) for home birth transfer. Antibiotic exposure was also higher among home-born neonates. When including all infants (recipients and non-recipients), the median number of antibiotic days was 2d (IQR 1-4) for home births compared with 1d (IQR 0-2) for both birth center and en route deliveries. Among infants who received antibiotics, the median duration was 2 days for both home (IQR 2-6) and birth center (IQR 1-3.2) and 2.5 days for those born en route (IQR 1.8-7). Among the 226 neonates in the cohort, 38 of 99 home-born infants (38.4 percent) required resuscitation compared with 44 of 110 infants delivered in birthing centers (40.0 percent). Respiratory indications accounted for 38 of 82 resuscitation events (46.3%), and neurologic concerns accounted for 20 of 82 events (24.4%). Together, these two categories represented 70.7% (58/82) of all resuscitation events.

Invasive support was also more common in the out-of-hospital birth population. Central line placement was required in 23.9% of out-of-hospital transfers (*n* = 54), with rates highest among home-born infants compared to those from birth centers. Ten neonates (4.4%) died prior to discharge, including six from home births, two from birth centers, and two born in transit. Among the 10 deaths, the primary causes of death were severe hypoxic-ischemic encephalopathy or other major neurologic injury (*n* = 7) and sepsis or septic shock (*n* = 3). Median survival time was 1 day (range: 0–40 days), with five deaths occurring on the day of birth and seven deaths occurring within the first 48 h of life (Table 1).

**Table 1:** Maternal and neonatal characteristics by birth setting.

Characteristic	Birth Center (*n* = 66)	Home (*n* = 53)	En Route (*n* = 13)
Length of Stay (days)			
n with available data	60	52	—
Mean	7.1	25.4	—
Median	5.0	11.5	**—**
IQR	3.0–8.0	5.0–31.5	—
Antibiotic Days — All Patients (including zeros for non-recipients)			
n (n receiving antibiotics)	66 (48)	53 (43)	13 (8)
Mean	2.3	4.4	2.6
Median	1.0	2.0	1.0
IQR	0.0–2.0	1.0–4.0	0.0–3.0
Antibiotic Days — Recipients Only			
n	48	43	8
Mean	3.2	5.5	4.3
Median	2.0	2.0	2.5
IQR	1.0–3.2	2.0–6.0	1.8–7.0

1 IQR=interquartile range (Q1 – Q3).

2 Length of Stay: individual patient values extracted from embedded formulas. Six birth center patients and one home patient had no LOS data recorded; en route group had no individual LOS data available.

3 Antibiotic Days (All Patients): zeros assigned to patients with no antibiotic exposure; denominator equals total group size.

4 Antibiotic Days (Recipients Only): restricted to patients who received at least one day of antibiotics.

5 En route group=deliveries occurring in transit (ambulance or car).

Survey responses received from 6 of 13 centers revealed substantial heterogeneity in infrastructure, eligibility criteria, and staffing among birth centers. All centers reported permitting waterbirths, and the average distance to the nearest hospital was 12 min, though individual centers ranged from 5 to 20 min. Delivery eligibility guidelines were broad: all centers (100%) accepted first-time mothers and patients with a history of cesarean delivery. All but one accepted people with pregnancies beyond 41 weeks. By contrast, only two centers (33%) allowed vaginal breech deliveries, and two (33%) permitted multiple gestations. Birth volume and discharge practices also varied. The average number of births per center was 154 annually. The average postpartum discharge time was 4.4 h.

Staffing patterns demonstrated similar variation. On average, centers reported 3.7 midwives, 1.5 nurses, and 1.5 medical assistants, for a mean total staff size of 6.8. At the time of delivery, however, most births were attended by a single provider, typically a certified professional midwife, with additional support from a student or birth assistant. None of the centers reported physician involvement in intrapartum care. Training and certification were not uniform. While all centers (100%) employed licensed midwives, only 83% had certified professional midwives, and one-third (33%) employed certified nurse-midwives. Five of six centers (83%) reported that Neonatal Resuscitation Program (NRP)-certified providers were present at every delivery; one center reported NRP-certified coverage at most but not all deliveries (Table 2).

**Table 2:** Birth center characteristics.

Center	Births per Year	Distance to Hospital (miles)	Discharge Time (hrs.)	Total Staff	Water Births Allowed	GBS Prophylaxis	Provider Training
Center #1	20	Variable	4	1	Yes	Not Required	CPM, LM
Center #2	60	5.6	3	6	Yes	Not Required	CPM, LM
Center #3	45	Variable	Not applicable	7	Yes	Not Required	CPM, LM
Center #4	200	3.5	5	7	Yes	Required	CPM, CNM, LM
Center #5	250	2.5	5	8	Yes	Required	CPM, LM
Center #6	350	1.0	5	12	Yes	Required	CNM, LM

Key: Key operational and clinical characteristics across participating birth centers, including annual delivery volume, average distance traveled for clients, staffing models, transfer policies, and credentialing requirements for midwives. The table highlights substantial variability in delivery volume, staffing structure, and transfer protocols among centers. Providers include Certified Professional Midwife (CPM), Licensed Midwife (LM) and Certified Nurse-Midwife (CNM).

GBS = Group B Streptococcus. Prophylaxis requirements reflect center policy at time of data collection.

Clinical protocols also varied. All centers used handheld Dopplers for intrapartum monitoring, but only two (33%) had access to continuous fetal monitoring, and none had fetal scalp electrodes. While GBS screening was universal, only two centers consistently administered intrapartum antibiotic prophylaxis when indicated, two provided antibiotics only upon request, and the remainder transferred patients for treatment. Intravenous catheter placement was available at only half the centers. Bag-mask ventilation equipment was universally reported, but suction devices were inconsistently available, and no center stocked defibrillators or advanced airway equipment. Transfer practices further highlighted variability. Some centers reported only one maternal or neonatal transfer per year, whereas others reported more than a dozen.

## Discussion

Neonates admitted to the NICU after out-of-hospital births had elevated morbidity, mortality, and healthcare resource utilization (23, 36). Home births were associated with longer NICU stays, greater antibiotic exposure, and higher rates of resuscitation compared with birth center deliveries. Significant variability existed among freestanding birth centers in staffing, emergency preparedness, clinical protocols, and transfer practices. Mortality was high (4.4%), with the majority of deaths attributed to severe hypoxic-ischemic encephalopathy or neurologic injury.

These findings are consistent with prior U.S. studies demonstrating increased neonatal risk associated with planned home birth, particularly in settings with limited integration into hospital systems (4–7, 11, 12). Our results extend these observations by providing contemporary, patient-level data from a regional Level IV NICU in Texas, a state with relatively low midwifery integration and variable regulatory oversight of birth centers (25). The higher severity observed among home birth transfers in our cohort, despite fewer overall admissions from this setting, may reflect delayed recognition of complications or longer transport times, as suggested by previous analyses of emergency transfers (9, 11).

Birth center preparedness and practice variability emerged as important modifiers of risk. Centers with lower transfer volumes tended to have more structured eligibility screening and written transfer protocols. Conversely, many centers lacked key capabilities recommended by ACOG and AAP, including consistent intrapartum antibiotic prophylaxis, continuous fetal monitoring, intravenous access, and guaranteed presence of NRP-certified providers at every delivery. These gaps align with national concerns about inconsistent emergency preparedness in freestanding birth centers (15, 16, 19) and support recommendations for minimum safety standards, particularly in regions with longer transport times (31).

Mortality in this cohort was driven predominantly by severe hypoxic-ischemic encephalopathy and sepsis. The predominance of neurologic injury as a cause of death is consistent with reports from other U.S. cohorts and state-level analyses linking out-of-hospital birth with increased rates of HIE (7, 8, 11, 30). The high proportion of deaths occurring on the day of birth or within the first 48 h underscores the critical importance of rapid recognition and transfer when complications arise.

Strengths of this study include the relatively large sample of neonates transferred after out-of-hospital delivery to a single regional Level IV NICU over five years, allowing detailed characterization of clinical outcomes and resource utilization. The combination of quantitative NICU data with survey data from local birth centers provides a more comprehensive view of system-level factors than studies relying solely on administrative datasets. As a major referral center, our institution likely captured the majority of high-risk transfers from the western Dallas–Fort Worth metroplex and surrounding areas during the study period (35).

Several limitations should be acknowledged. First, this study captured only neonates admitted to a Level IV NICU and therefore represents a high-risk subset; infants with adverse outcomes who died before transfer, were managed in emergency departments, or were admitted to lower-acuity units were not included, likely resulting in overestimation of morbidity and mortality. Second, the survey of birth centers had a modest response rate (46%), and voluntary participation may have introduced selection bias if centers with poorer infrastructure or higher complication rates were less likely to respond. Third, classification of births as planned vs. unplanned was limited by inconsistent documentation in transfer records. Fourth, outcome definitions relied on documentation at the time of transfer and admission, which may have introduced misclassification. Finally, the single-center design and focus on Texas limit generalizability to other states with different regulatory environments, midwifery integration, and healthcare infrastructure.

In conclusion, neonates transferred to a Level IV NICU after out-of-hospital birth experienced high rates of morbidity, mortality, and resource utilization, with home births demonstrating greater risk than birth center deliveries. Substantial variability in birth center preparedness and practice patterns was observed. These findings highlight the need for stronger regulatory standards, improved emergency preparedness, and better integration of community birth settings into regional perinatal systems (32, 33). Further research is needed to evaluate the impact of specific regulatory and practice interventions on neonatal outcomes and to develop standardized reporting frameworks for out-of-hospital births.

## Data Availability

The raw data supporting the conclusions of this article will be made available by the authors, without undue reservation.
