# Supplementary material for: Out-of-hospital births and neonatal outcomes: a retrospective cohort study from a level IV NICU and review of regulatory frameworks
Source: Front Pediatr. 2026 Aug 28;14:1843232. doi: 10.3389/fped.2026.1843232 (PMC13562055; doi:10.3389/fped.2026.1843232)
Supplement: Supplementary file 1 [file Datasheet1.pdf]

Birthing Centers in Tarrant County

Facility Survey & Assessment Questionnaire

SECTION 1 — FACILITY INFORMATION

**Name of Facility**	
**Address**	
**Date Opened**	
**Total Staff**	

SECTION 2 — STAFFING

Staff Category	Number
Midwives	
Nurses	
Medical Assistants (if applicable)	
Other (please specify below)	

SECTION 3 — SERVICES PROVIDED

What services does your facility provide?

■ Prenatal Care	■ Labor and Delivery	■ Postpartum Care
■ Lactation Support	■ Newborn Care	■ Other

Please describe any additional services:

SECTION 4 — PATIENT DEMOGRAPHICS

Select all racial/ethnic groups that best represent the population at your birthing center:

■ White	■ African American	■ Hispanic / Latino
■ Asian	■ Pacific Islander	■ Multiracial
■ Other		

SECTION 5 — ANNUAL DELIVERIES

How many babies were delivered at your facility each year?

2020	2021	2022	2023	2024

SECTION 6 — CARE PROVIDERS & TRAINING

Training and certification level of primary care providers for laboring women:

How many healthcare providers attend each delivery?

What staff are present at each delivery?

Who is present to care for the infant?

Is the same person caring for the mother also caring for the infant?	**Y / N**
Is this person NRP certified?	**Y / N**

SECTION 7 — LENGTH OF STAY & DISCHARGE

Duration of stay (typical range)

Median time for discharge after birth

Policies/procedures for mother & baby discharge

SECTION 8 — DELIVERY ELIGIBILITY CRITERIA

Does your center have a policy or guideline regarding criteria to deliver at your facility?

Pregnant patients without a previous delivery	**Y / N**
Pregnant patients at 41 weeks' gestation or more	**Y / N**

Fetal malpresentation (breech or other)	**Y / N**
Previous C-section	**Y / N**
Multiple gestation	**Y / N**

SECTION 9 — WATER BIRTHS & DELIVERY OPTIONS

Are there water births at your facility?	**Y / N**
Can patients deliver in a bathtub?	**Y / N**
Can patients deliver in a shower?	**Y / N**

How far (in minutes) is the nearest hospital that accepts NICU patients?

____

SECTION 10 — INTRAPARTUM MONITORING TOOLS

■ Fetal Heart Rate Tracing	■ Fetal Scalp Electrode
■ Intermittent Auscultation	■ Ultrasound Capabilities

SECTION 11 — GBS SCREENING & INFECTION MANAGEMENT

Do you screen for GBS colonization?	**Y / N**
Do you treat with antibiotics during labor for GBS-colonized women?	**Y / N**
Do you treat with antibiotics during labor for women with chorioamnionitis?	**Y / N**

SECTION 12 — INFANT CARE SERVICES

Of the options below, what infant care services do you provide?

■ Newborn exam with APGAR scores	■ Monitoring for early-onset sepsis	■ Glucose screening
■ Eye prophylaxis	■ Vitamin K	■ Hepatitis B vaccine
■ Screening for hyperbilirubinemia	■ State newborn screen	■ Hearing screening
■ Pulse oximetry screening	■ Congenital heart screening	■ Car seat testing

SECTION 13 — RESUSCITATION EQUIPMENT

Infant Resuscitation Tools Available:

■ Supplemental O■	■ Wall Suction	■ Bag-Mask Ventilation
■ Radiant Warmer	■ Umbilical Catheters	■ Endotracheal Tubes

Maternal Resuscitation Tools Available:

■ Supplemental O■	■ Bag-Mask Ventilation	■ Laryngoscope
■ Endotracheal Tubes	■ Mechanical Ventilator	■ CPAP
■ Cardiac Monitor / Defibrillator	■ Automated External Defibrillator (AED)	■ Epinephrine & Resuscitation Medications
■ Peripheral IV Catheter	■ Blood Products	

SECTION 14 — TRANSFER CRITERIA

What are your indications that a patient and/or their child should be transferred to a hospital?

What is your criteria to refer a mother to deliver at a hospital instead of remaining at your facility?

SECTION 15 — ANNUAL TRANSFER DATA

Mothers referred to hospital before delivery:

2020	2021	2022	2023	2024

Mothers referred to hospital after delivery:

2020	2021	2022	2023	2024

Infants referred to hospital:

2020	2021	2022	2023	2024

SECTION 16 — FACILITY TOUR

Would you be willing to have a member of our team tour your facility?

■ Yes, please contact us	■ No, thank you	■ Maybe — contact us to discuss

Best contact name and phone/email:

Thank you for taking the time to complete this survey. Your responses will help improve maternity care resources in Tarrant County.
